# In vitro culture of bovine preantral follicles: a review

**DOI:** 10.1186/1477-7827-12-78

**Published:** 2014-08-13

**Authors:** Valdevane R Araújo, Melba O Gastal, José R Figueiredo, Eduardo L Gastal

**Affiliations:** Department of Animal Science, Food and Nutrition, Southern Illinois University, 1205 Lincoln Drive, MC 4417, Carbondale, IL 62901 USA; Laboratory of Manipulation of Oocytes and Preantral Follicles (LAMOFOPA), Veterinary Faculty, State University of Ceará, Av. Paranjana 1700, Campus do Itaperi, Fortaleza, CE 60740-903 Brazil

**Keywords:** Cow, Folliculogenesis, Oocyte, Ovarian follicles

## Abstract

Preantral follicles are the majority of the ovarian follicle population and their use as a source of homogeneous oocytes for bovine reproductive biotechnologies could result in a substantial advance in this field. However, while in other species embryos and offspring have been produced, in bovine species the results have been limited to the follicular activation of small (primordial) preantral follicles and formation of early antral follicles from large (secondary) preantral follicles after in vitro culture. Therefore, this review will highlight the basic aspects of bovine folliculogenesis by focusing on preantral follicles, the methods of harvesting preantral follicles, the main results from in vitro follicular culture during the last 20 years, and the potential candidate substances (basic supplements, growth factors, and hormones) for improving the efficiency of in vitro follicle growth.

## Background

The mammalian ovary is responsible for the development, maturation, and release of mature oocytes for fertilization, as well as for the synthesis and secretion of hormones that are essential for follicular development, menstrual/estrous cyclicity, and maintenance of the reproductive tract and its function. In cattle, from mid-pregnancy to reproductive senescence many follicles are activated to enter the growth phase, which is characterized by both proliferation of the granulosa cells and an increase in the oocyte size [1]. However, most of these follicles gradually become atretic during in vivo growth phase; this fact has stimulated great interest in the development of a culture system that might be able to maintain follicular growth and avoid this loss of follicles.

Considering that primordial follicles constitute the supreme starting material for in vitro culture due to their large number when compared with mature follicles [2], it would be of remarkable help to possess a renewable source of primordial follicles from high-yielding animals for culture in order to maximize offspring from these animals [3]. Moreover, elucidation of the poorly understood mechanisms of primordial follicle activation would constitute an important leap forward in the understanding of follicular dynamics [4].

Preantral follicles for research are usually obtained from ovaries from slaughterhouses or through laparotomy or ovarian biopsies. Studies using ovarian biopsy have shown minimum or no disturbance to ovarian function in several species, including cattle [3–5], horses [6–8], and humans [2, 9]. This technique will be of great value for experimental or diagnostic purposes. Profound similarities in the dynamics of follicle development exist between the menstrual cycle in women and the estrous cycle in cattle and horses [10, 11]. In this regard, research using animal models for studying human ovarian function is important to provide a hypothetical basis for further studies in women, which will ultimately lead to the development of safer and more efficacious infertility and contraceptive therapies [12]. Therefore, if preantral follicles could be efficiently isolated from ovaries, a large potential source of oocytes (genetic material) could be obtained to reach meiotic competence in vitro. Moreover, immature oocytes from preantral follicles could be used in other assisted reproductive technologies, such as in vitro maturation and embryo production, transgenesis, and conservation of endangered species.

An in vitro follicle growth system that allows complete growth of oocytes from preantral or early antral follicles has been studied. However, besides the differences among species, in vitro follicle growth success depends on initial oocyte size, as well as follicle categories used. In regard to large animals, the production of embryos from buffalo [13], sheep [14, 15], goats [16], and monkeys [17] has been obtained only from large advanced secondary follicles. However, in mice, embryos and live offspring have been produced using developmentally competent oocytes obtained after growth entirely in vitro starting with primordial follicles [18, 19]. In bovine species the best results have been the antrum cavity formation (tertiary follicles) after in vitro culture of advanced secondary follicles [20].

Ovarian follicular development and oocyte growth depend on a bidirectional communication between oocytes and somatic cells. Oocytes have an essential role in controlling the proliferation and differentiation of granulosa cells during follicular development [21]. The ability to sustain preantral follicle growth in vitro to support the acquisition of oocyte competence could represent a breakthrough in the reproduction field since this source of oocytes could be beneficial for assisted reproductive technologies. Additionally, research aiming at further understanding of somatic cell-oocyte interactions in species with prolonged follicular growth, such as bovines [22], would be of great significance for human reproduction. Therefore, in vitro culture systems have to allow for these conditions and properly maintain cell interactions during follicle development.

This review aims to describe and discuss the advancements in and current status of the emerging research with bovine preantral follicles. Firstly, we summarize current knowledge of achievements in the development of in vitro systems for culture of bovine preantral follicles. Secondly, we address the methods of harvesting preantral follicles, the culture media, and the systems used. Finally, we describe the most common growth factors and hormones utilized to culture bovine preantral follicles.

## Review

### Basic aspects of early bovine folliculogenesis

**Figure 1:**
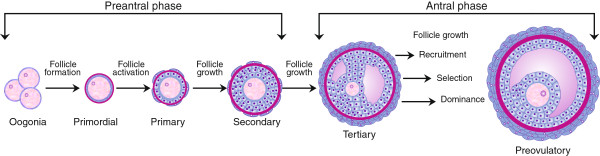
**Schematic sequence of complete follicular development.** Preantral phase: Formation and beginning of growth and activation of primordial follicles and growth of primary and secondary follicles. Antral phase: formation of tertiary follicle (antral-filled follicular fluid cavity). Follicle growth continues through the phases of recruitment, selection, dominance, and preovulatory stage of follicular waves. Oogonia develop from a primordial germ cell and differentiates into an oocyte in the ovary. Primordial follicle has a single layer of flattened granulosa cells. Primary follicle has a single layer of cuboidal granulosa cells. Secondary follicle has two or more layers of cuboidal granulosa cells and a small number of theca cells. All the preantral follicles have a primary oocyte. Tertiary follicle has several granulosa cell layers, theca cells, and primary oocyte and is characterized by an antral cavity which contains follicular fluid. Preovulatory or also called as Graafian follicle is the last stage of follicle development; these follicles are larger, have more antral fluid and may contain a secondary oocyte. Follicular fluid is a plasma exudate conditioned by secretory products from the granulosa cells and oocyte.

Development of bovine oocytes and follicles begins in the fetal phase [23] and takes around 6 months to be completed [24]. The follicle development is comprised of two distinct and consecutive phases (Figure 1): the first phase, characterized by the formation and beginning of growth of primordial follicles, and the second phase, in which the growth of primary and secondary follicles occurs as granulosa cells transform from a flattened to a cuboidal shape and proliferate, while the oocyte experiences a rapid increase in size. It has been reported that the critical point of follicle growth is when the follicle has about 40 granulosa cells and the oocyte undergoes the first significant change in diameter [25].

#### Formation and initiation of primordial follicle growth

Primordial germ cells proliferate by mitosis to form primary oocytes with the first meiotic prophase starting between days 75 and 80 of pregnancy in cattle [26]. The formation of primordial follicles occurs at the pachytene stage of meiosis, between days 91 and 144 of pregnancy [27] and then arrest at the diplotene stage [27, 28]. In primordial follicles the oocyte is surrounded by a single layer of six pre-granulosa (flattened) cells, which is in turn surrounded by a basal membrane; these are the first generation of follicle cells [25] and are derived from the celomic epithelium. From day 170 onward, the ovigerous cords of primordial germ cells are absent and there are only primordial follicles present [26]. After the formation of primordial follicles, the pre-granulosa cells stop multiplying and remain in the resting phase until they are stimulated to grow [26]. In bovine species, primordial follicles have a mean diameter of 35.2 μm and oocyte growth is initiated only during the fourth generation of follicle cells, compared with the second or third generation in rodents and humans, respectively [25].

During the initiation of follicular growth, in a phase known as primordial follicular activation, some primordial follicles leave the reserve pool of quiescent follicles to enter the growing pool (transitional, primary, secondary, tertiary, and preovulatory stage [23]. The activation of primordial follicles is a nonreversible process; therefore, it is important in regulating the size of the resting primordial follicle pool, which will affect the reproductive lifespan and fertility [27]. Follicular activation is characterized by the morphological modifications of granulosa cells from flattened to cuboidal, as well as the resumption of cell proliferation [29] and the initiation of oocyte growth. However, the factors and mechanisms responsible for the control of early folliculogenesis are still poorly known and represent one of the major questions related to ovarian biology.

#### Growth of primary and secondary follicles

After activation, bovine primordial follicles gradually acquire cuboidal granulosa cells and become transitional and primary follicles, the latter with one complete layer of 11-40 cuboidal granulosa cells around the oocyte observed in the largest cross section of the follicle [25, 30]. Secondary follicles are characterized by the addition of a second complete layer of granulosa cells, the initial deposition of zona pellucida (ZP) material, formation of cortical granules within the oocyte cytoplasm [31], the beginning of theca cell layer formation [25], mRNA synthesis in the oocyte [22], and gonadotropin responsiveness [32].

Primary and secondary follicles appear in the bovine fetal ovary around days 140 and 210 [23], and have a mean diameter of 46.1 μm [30] and 81.0 μm (early secondary follicles; [25]), respectively. Unlike in primordial follicles, at these follicular stages the ZP begins to form, surrounding the oocyte [31, 33]. Braw-Tal and Yossefi [25] verified that the ZP first appeared in early secondary follicles (range, 81-130 μm in diameter), but formed a complete ring around the oocyte during the late secondary follicle stage (range, 131-250 μm in diameter).

The growth of preantral follicles after the primary stage also depends on important events that include the expression of growth and differentiation factors such as vascular endothelial growth factor (VEGF) and growth and differentiation factor-9 (GDF-9). VEGF, in particular, has been considered as a stimulator of bovine follicular development in vitro because it provides support for the transition from the primary to the secondary follicle stage [34].

During growth of secondary follicles an organization of the granulosa cells occurs in several layers and an antral cavity filled with follicular fluid is formed among these cells [31]. From this stage onwards, the follicles are called tertiary or early antral follicles and have been observed during the bovine fetal phase at days 210 [35] or 230-250 [23] of gestation. The transition from secondary to tertiary stage includes the development of the internal and external theca cell layers and the beginning of cumulus cell formation [31] in follicles around 120 μm in diameter [24].

Although antral cavities are usually established when the follicles reach at least 200 μm in diameter [24, 36], as we mentioned previously, large secondary follicles (greater than 190 μm in diameter) have been mechanically isolated from bovine ovaries [37–40], as well as from the ovaries of other species such as goats [16, 41, 42] and sheep [14, 15].

### Harvesting bovine preantral follicles

#### Mechanical isolation using tissue chopper or microdissection

The first studies using mechanical isolation techniques were developed during the early 1990s and represented major advances in the isolation of morphologically normal preantral follicles. Early preantral follicles were mechanically isolated using a machine called a tissue chopper [43], a homogenizer [44], or a cell dissociation sieve [45, 46]. Furthermore, isolation of later stage preantral follicles via microdissection was reported using insulin needles [47].

**Figure 2:**
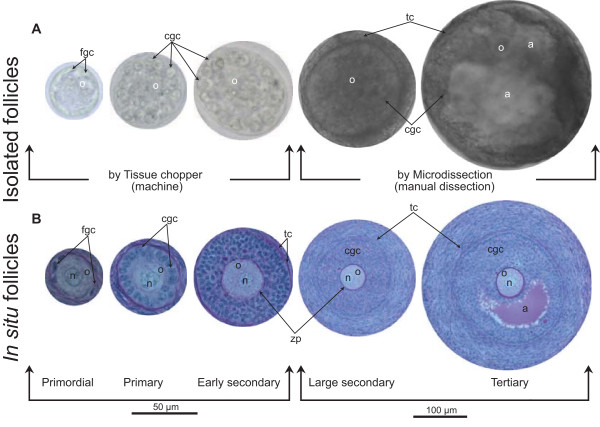
**Isolated follicles by tissue chopper and microdissection, and** ***in situ*** **follicles.** Isolated follicles **(A)** by tissue chopper and microdissection, and **(B)** *in situ* follicles stained with PAS-hematoxylin. o: oocyte; n: oocyte nucleus; fgc: flattened granulosa cells; cgc: cuboidal granulosa cells; tc: theca cells; zp: zona pellucida. A large secondary follicle and an antral follicle grown in vitro and isolated by microdissection are shown **(A)**.

Bovine preantral follicles have been successfully isolated utilizing tissue chopper and microdissection. Both follicular isolation methods have shown no detrimental effect on the tridimensional structure of the small follicles because they did not damage the basal membrane ([43, 48]; Figure 2A). The preservation of the follicular basal membrane may prevent the spreading of granulosa cells during in vitro culture [48], preserving follicular morphology by maintaining follicular adhesion to extracellular compounds. Additionally, the basement membrane contains proteoheparansulfate [49], which binds to a variety of growth factors [50]. Therefore, the presence of a basement membrane around the follicles might optimize the effects of growth factors and hormones added to the culture medium [51].

The number of follicles isolated by tissue chopper differs according to the species [52] and even among breeds. In goats and sheep, the best results were obtained with the intervals of 75 and 87.5 μm, respectively [53, 54]. The best interval for sectioning ovarian tissue varies from 50 μm for European cattle (*Bos Taurus*; [43] and 125 μm for Zebu cattle (*Bos Indicus*; [52]. These differences regarding the cut interval most suitable to isolate preantral follicles may be explained by differences in quantity of ovarian tissue and variation in its composition, as in corpora lutea and corpora albicans [43]. Follicles embedded in a more fibrous stroma can be more difficult to isolate and smaller cut intervals would be necessary [52].

The microdissection method has been used to isolate large bovine follicles using fine needles under stereomicroscopy. This method maintains the theca cell layers, which ensures follicle quality [55, 56]. In general, the presence of theca cells is a crucial condition for normal follicular growth, preservation of estrogen production [57], maintenance of follicular health, and remodeling of the basement membrane [58]. Therefore, maintaining communication among the oocyte, the surrounding somatic cells, and the extracellular matrix is vital to the achievement of normal folliculogenesis, and to sustain follicular growth and viability [58]. This technique allows the isolation of several morphologically normal and intact follicles from ovarian tissue. Large bovine preantral follicles have been successfully isolated and cultured in vitro until antrum formation after short- [22, 58] and long-term [20, 37–39, 59] culture.

#### Enzymatic isolation

The fibrous nature of the ovaries of most domestic species complicates follicular isolation [60]. Therefore, some studies have been conducted using different types of enzymes to recover preantral follicles in different species. Collagenase (from Clostridium histolyticum) has been used to isolate numerous preantral follicles from murine [61], swine [62], and bovine [43] ovaries. In addition, an enzymatic method using deoxyribonuclease (DNAse) has been developed to isolate human follicles [63]. However, this latter process requires a lengthy cooling time and consequently reduces the viability of the follicles by causing damage to cell membranes.

The degree of enzymatic damage depends on the duration of treatment, the concentration of the enzyme(s), and the type of tissue [43, 64]. Morphologically normal follicles have been isolated from bovine ovaries using a combination of collagenase and DNAse treatment [65]. However, it was reported that although the oocytes from freshly isolated preantral follicles appeared healthy under an inverted microscope, histological examinations revealed that the enzymatic process could have damaged the oocytes, especially in smaller preantral follicles.

#### Ovarian biopsy in vivo

A new method for the repeated collection of ovarian biopsies from living donors through transvaginal, ultrasound-guided puncture of the ovary has been successfully developed and tested in cows [3], women [66], and recently in mares [6–8]. This procedure can be seen as a modified version of a commercial ovum pick-up (OPU) technique. Using ovarian biopsies, Aerts et al. [3] had an average success rate of 68% for recovering ovarian fragments. Although rather small, these fragments were suitable for both histological (Figure 2B) and immunohistochemical evaluation and revealed the presence of morphologically normal primordial and growing preantral follicles. In addition, in a later study, the restoration of ovarian tissue morphology (using light microscopy) and the preservation of follicle viability (using fluorescence microscopy) in the majority of preantral follicles after multiple ovarian biopsy sessions was reported [4].

### In vitro culture systems for bovine preantral follicles

**Figure 3:**
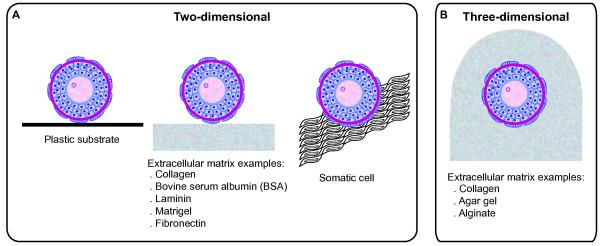
**Schematic representation of the two- and three-dimensional culture systems.** Schematic representation of the **(A)** two- and **(B)** three-dimensional culture systems utilized for bovine preantral follicles.

For bovine and other farm domestic species, the development of culture systems capable of supporting the growth of immature follicles to a stage where they could be matured and the oocyte fertilized would ensure a large supply of oocytes for manipulation. These oocytes could potentially be used to shorten the generation interval of selected animals and, consequently, to increase the number of offspring born per animal. Development of a successful culture system for preantral follicles with immature oocytes is dependent upon efficient procedures to recover the follicles from the ovary and culture them as well.Basically, there are two ways to culture bovine preantral follicles: 1) enclosed in ovarian tissue fragments (slices or strips), also called “in situ”; or 2) using isolated follicles. Isolated follicles have been cultured either in a two-dimensional (2D) system (Figure 3A) – i.e. the follicle is placed on the surface, which may be a plastic or extracellular matrix (e.g., collagen gel, matrigel, etc) – or in a three-dimensional (3D) system (Figure 3B), in which the follicles are cultured within an extracellular matrix.

Currently, the major use of isolated follicles for culture is to support the growth and development of immature oocytes and allow the understanding of the mechanisms involved in oocyte development, granulosa cell differentiation, and regulation of autocrine/paracrine factors that control early stages of folliculogenesis [67].

#### In vitro culture of preantral follicles enclosed in ovarian tissue (in situ)

In the in situ culture system, follicles are cultured with the surrounding ovarian tissue, including the stromal cells. This culture system allows the interaction between the follicles and their adjacent cells, such as stromal/theca cells and granulosa cells, which may influence their growth [68]. This is a very practical method and prevents prolonged exposure of the cells to the external environment.

The spontaneous activation of primordial follicles has been known to occur in vitro using the in situ culture system in several species, including mice [18, 69], cattle ([25, 70–74]; Table 1), horses [7], goats [75, 76], and primates [77, 78]. The majority of the bovine primordial follicles cultured in situ may activate within 2 days of culture [25, 27, 70] and reach the secondary stage in 6 [79], 10 [27, 34, 80], or 22 days [74].

**Table 1:** Chronological advances in in situ culture system of early bovine preantral follicles*

Authors	Duration of culture (days)	Type of medium utilized	Maintenance of follicular survival and/or viability	Follicular activation (from primordial to transitional or primary stage)	Increase of follicular and/or oocyte diameter
**Peluso and Hirschel, 1988**	2	TCM-199	Yes	Yes	Yes
**Wandji et al., 1996a**	0, 2, 4, or 7	Waymouth	Yes	Yes	Yes
**Braw-Tal and Yossefi, 1997**	4	α-MEM	-	-	Yes
**Fortune et al., 1998**	7 or 14	Waymouth	Yes	Yes	Yes
**Derrar et al., 2000**	8	Waymouth, α-MEM	Yes	Yes	-
**Gigli et al., 2006**	7	Waymouth	-	Yes	Yes
**Yang and Fortune, 2006**	10	Waymouth	-	Yes	Yes
**Yang and Fortune, 2007**	10	Waymouth	-	Yes	Yes
**Yang and Fortune, 2008**	2 or 10	Waymouth	-	Yes	-
**McLaughlin and Telfer, 2010**	6	McCoy’s	Yes	Yes	Yes
**Andrade et al., 2012**	8	α-MEM	Yes	Yes	-
**Tang et al., 2012**	22	α-MEM	-	Yes	Yes

*All the results were compared with the fresh control group (Day 0).

Despite the fact that in vitro culture of ovarian tissue is able to develop primordial follicles until primary and secondary stages [27, 34, 74, 80], this technique has not been very effective for follicle maturation. A two-step culture system for bovine [79] and human [81] preantral follicles has been tested recently. The aim of this system was to determine whether in situ-grown bovine and human follicles could be isolated at the secondary stage and cultured to late preantral/early antral stages. However, in both species, only a few antral follicles were obtained after 4 [81] or 15 days [79] of culture of secondary follicles.

#### In vitro culture of isolated preantral follicles

Although primordial and primary follicles can easily be isolated from bovine ovaries using mechanical or enzymatic methods, mostly small (diameter ≤150 μm) and large (diameter >150 μm) secondary follicles have commonly been used for this in vitro culture system [37–40, 79]. In the bovine species, several studies have used in vitro culture of isolated follicles (Table 2). The best results produced so far have been obtained from culture of large secondary follicles [22, 37–40]. It has been reported that early secondary follicles (75 to 125 μm in diameter) attached to the culture wells and created a monolayer, which resulted in flattened and damaged follicular structures [55]. Conversely, the culture of isolated large secondary follicles was able to maintain follicular viability, increase follicular diameter, and foster estradiol and progesterone production [65].

**Table 2:** Chronological advances in two and three dimensional (2D and 3D) in vitro culture systems for isolated bovine preantral follicles*

Authors	Duration of culture (days)	Follicular diameter (μm) ^†^	Type of medium utilized	Culture system description	Maintenance of follicular survival and/or viability	Increase of follicular and/or oocyte diameter	Antrum formation	Steroid secretion
Figueiredo et al., 1994a	5	30-70	α-MEM	2D-plastic substrate (3 follicles/drop)	Yes	Yes	-	-
Figueiredo et al., 1994b	5	30-70	α-MEM	3D-collagen (4 follicles/well)	Yes	Yes	-	-
Figueiredo et al., 1995	1	30-70	α-MEM	2D-uncoated plastic or coated with BSA, Laminin, Fibronectin, Matrigel, or Collagen and 3D-Collagen	-	-	-	-
Wandji et al., 1996b	6	60-220	Waymouth	3D-agar gel (30-40 follicles/drop)	Yes	Yes	-	E2 and P4
Hulshof et al., 1997	5	30-70	α-MEM	3D-collagen (5 follicles/drop)	Yes	Yes	-	-
Schotanus et al., 1997	8	30-80	TCM-199	3D-collagen (5-10 follicles/drop)	Yes	Yes	-	-
Katska and Rynska, 1998	23	75-195	TCM-199, Menezo B2	2D-under mineral oil	Yes	Yes	-	-
Gutierrez et al., 2000	28	166 ± 2.2	McCoy’s	2D-plastic substrate (1 follicle/well)	-	Yes	Yes	-
Itoh and Hoshi, 2000	30	30-70	TCM-199	2D-somatic cells (15-20 follicles/well)	Yes	Yes	-	-
McCaffery et al., 2000	6	100-200	McCoy’s	2D-plastic substrate	-	Yes	Yes	-
Saha et al., 2000	10	40-100	TCM-199	2D (1-3 follicles/well)	Yes	Yes	-	-
Thomas et al., 2001	12	146 ± 1.7	McCoy’s	2D-plastic substrate	-	Yes	-	E2
Itoh et al., 2002	13	145-170	TCM-199	3D-collagen	-	Yes	Yes	E2
Saha et al., 2002	7	120	TCM-199	2D	Yes	Yes	No	-
Thomas et al., 2007	6	145 ± 0.6	McCoy’s	2D	-	Yes	No	E2
McLaughlin et al., 2010	8	157 ± 3	McCoy’s	2D	-	Yes	Yes	-
McLaughlin and Telfer, 2010	12-15	111 ± 1.5	McCoy’s	2D	-	Yes	-	E2
Rossetto et al., 2012	16	>150	α-MEM, TCM-199, McCoy’s	2D-plastic substrate	Yes	Yes	Yes	-
Rossetto et al., 2013	18	>150	α-MEM	2D-plastic substrate	Yes	Yes	Yes	-

*All the results were compared with the fresh control group (Day 0). Estradiol (E2) and Progesterone (P4) hormones.

^†^At Day 0 of culture.

In general, isolated bovine preantral follicles have been cultured using well plates without mineral oil in a 2D culture system. In vitro culture without mineral oil has maintained follicular viability and increased antrum formation [20, 22, 39, 40] and estradiol production [22] in isolated bovine preantral follicles. Similarly, studies using mineral oil in a 2D culture system also have shown high follicular viability and antrum formation [37, 38, 55], as well as an increase in estradiol production after in vitro culture [38].

A 3D culture system has been developed to culture isolated preantral follicles in mice [82–84], buffalo [85], cows ([65, 86, 87], Araújo et al., unpublished observations), and human [88] and nonhuman [17] primates. In this method, the follicles are cultured inside an extracellular matrix, which mimics the ovary and maintains the spherical morphology of the ovarian follicle and preserves the cell-cell and cell-matrix connections important for regulating follicle development in vivo [89–91]. The 3D culture system maintained high follicular viability and increased follicular diameter [65, 86] and steroid production after in vitro culture [65]. It is believed that 3D systems more effectively simulate physiological conditions because many cellular processes in organogenesis occur exclusively in three dimensions [82].

### Improving in vitro growth of bovine preantral follicles

Oocyte-secreted paracrine factors promote the proliferation, differentiation, and function of granulosa cells. Moreover, the development of the oocyte in vitro to a stage where normal embryonic development can be supported is dependent on the oocyte reaching the appropriate stage of development to respond to the endocrine and paracrine signals responsible for the induction of maturation [92]. Therefore, an elucidation of the bidirectional interplay between these two cell types is also important for the development of a successful culture system [4].

#### Culture media

Different commercial media have been used to culture bovine preantral follicles in vitro (Tables 1 and 2). However, based on literature reports there has been no standard, reliable culture medium for bovine preantral follicles. The most commonly used culture media are: α-MEM [37–40, 48], TCM-199 [39, 55, 56, 93, 94], and McCoy [20, 22, 39, 58, 79, 95, 96]. The lack of standardized protocols may affect in vitro follicle culture and can also explain the different results from several research groups. Among the commercial culture media, TCM-199 and α-MEM have been the most commonly used to maintain follicular survival and viability and to improve the development of bovine follicles. A recent study compared TCM-199, McCoy, and α-MEM under the same experimental conditions and demonstrated that TCM-199 was the best medium to culture bovine secondary follicles, based on the high percentage of viable follicles after in vitro culture [39]. However, this study also revealed that follicles cultured in α-MEM or TCM-199 preserved at the ultrastructural level the cytoplasmic membrane and oocyte nucleus, and normally and uniformly distributed mitochondria and endoplasmic reticulum. Comparing α-MEM with TCM-199 we found recently that α-MEM can be used to replace TCM-199 for bovine preantral follicle culture if only progressive addition of medium without medium removal is used for medium change (Araújo et al., unpublished observations). These results provide new perspectives in order to identify the best culture system for each species, taking into consideration the base culture medium, supplements (hormones and growth factors), and medium replacement methods.

#### Basic supplements

Substances such as pyruvate, glutamine, hypoxanthine [48], and ascorbic acid [95] have been used with success for the culture of bovine ovarian preantral follicles. Therefore, these substances have become part of the base medium used to culture ovarian follicles of several species. Pyruvate and glutamine are energy substrates and the addition of both substances to the culture medium increased the percentage of intact follicles [94]. Pyruvate was shown as a predominant substrate used by immature bovine oocytes [97] and isolated growing mouse oocytes [98]. Glutamine is an efficient energy substrate required for biomass synthesis [99] by bovine preantral follicles [48]. Hypoxanthine is a substance that has increased the number of morphologically normal oocytes [48], maintained oocyte-granulosa cell communication during the culture of mouse preantral follicles [61], prevented meiotic resumption [100], and promoted oocyte growth in vitro [101]. It has been suggested that hypoxanthine improves the utilization of additional energy substrates by maintaining interactions between the oocyte and the surrounding granulosa cells [48].

Another important substance that has been used in culture media is ascorbic acid. Ascorbic acid is a vitamin that acts as an antioxidant and is involved in processes of hormone secretion, gonadal tissue remodeling, and apoptosis [102]. It has also been associated with several processes during follicular and luteal development [95] because it accumulates in granulosa cells, theca interna cells, luteal cells, and oocytes [103–105]. Moreover, it was observed in vitro that ascorbic acid maintained follicle integrity in the absence of serum, reduced the incidence of cell death, and may participate in the regulation of extracellular matrix remodeling by increasing matrix metalloproteinases-9 (MMP-9) activity [95]. Additionally, the addition of ascorbic acid to the culture medium stimulates the activation of in vitro cultured primordial follicles in cattle and subsequent growth of activated follicles [106]. Therefore, the use of ascorbic acid is crucial for culture of isolated follicles because it ensures the integrity of the basement membrane of the follicles.

#### Growth factors

Ovarian follicular growth is controlled by complex interactions between the oocyte and the surrounding granulosa and theca cells, as well as by locally produced growth factors and hormones. In addition, the balance of stimulatory and suppressive factors dramatically affects the growth of granulosa cells of small preantral follicles in vitro [65]. Among the known major growth factors present in bovine ovarian cells are insulin-like growth factors (IGFs; [96]), fibroblast growth factors (FGFs; [107, 108]), vascular endothelial growth factors (VEGFs; [34]), bone morphogenetic proteins (BMPs), and growth and differentiation factors (GDFs; [74, 109]). The following sections will describe only the growth factors which have been used in in vitro culture systems for bovine preantral follicles.

#### Insulin-like growth factor-1 (IGF-1)

The IGF-1 binding ontogeny [110] and its type 1 receptor mRNA have been demonstrated in the oocytes and granulosa and theca cells of bovine preantral follicles [111]. In addition, IGF binding protein-2 (IGFBP-2) mRNA has been detected in granulosa cells and oocytes, and IGFBP-3 mRNA in oocytes from bovine preantral follicles [112]. During the stages of antral and preovulatory follicles, IGF-1 mRNA has been detected in bovine granulosa [113] and theca cells [111], indicating that IGF-1 is important during the later stages of folliculogenesis (e.g., in relation to LH responsiveness [10].

IGF-1 has been identified as a stimulatory growth factor for bovine follicular and oocyte growth, as well as for antrum formation during prolonged culture [20, 59]. Long-term in vitro culture may allow the differentiation of granulosa cells by IGF-1, which acts as a stimulator of follicular development. Follicular growth [96], antral cavity formation [40, 58, 96], and estradiol production were observed after using IGF-1 in the in vitro culture of bovine preantral follicles. Conversely, McCaffery et al. [58] observed that treatment of immature follicles with IGF-1 resulted in precocious differentiation, which might have retarded follicular growth and cell proliferation. Recently, Rossetto et al. [40] showed that addition of IGF-1 to the culture medium of bovine preantral follicles had no effect on the follicular morphology and antrum formation. Similarly, we observed that IGF-1 did not interfere in any end point, including the estradiol concentrations, evaluated during the in vitro culture of bovine secondary follicles (Araújo et al., unpublished observations). Therefore, the action of IGF-1 in bovine oocyte and follicular development is strictly regulated by the developmental stage, period of culture, and concentration of IGF-1 used [114].

#### Basic fibroblast growth factor (bFGF)

Immunoreactivity, bioactivity, and mRNA of bFGF are present in bovine granulosa cells [115]. Both bFGF alone and bFGF in combination with FSH allowed the maintenance of follicular survival, promoted in vitro growth of granulosa cells, and increased the diameter of bovine preantral follicles. However, when bFGF was combined with transforming growth factor β (TGF-β), there was an inhibition of the stimulatory effect of bFGF on follicular diameter and a decrease in follicular survival [115]. Although bFGF alone stimulated estradiol and progesterone production during in vitro culture of bovine preantral follicles, it suppressed FSH-stimulated progesterone production [65]. These results suggest that bFGF antagonizes, at least in some aspects, the FSH-mediated cytodifferentiation of cultured bovine preantral follicles.

#### Vascular endothelial growth factor (VEGF)

VEGF has been known as a regulator of the various phases of follicle development [116]. Yang and Fortune [34] demonstrated that the mRNA for both VEGF receptors (flt-1 or VEGFR-1 and flk-1 or VEGFR-2), as well as for the VEGF ligand, were expressed in the fetal bovine ovary at day 90 of gestation. However, mRNA expression for the VEGF ligand increased when the first secondary follicles were observed at day 210 of gestation [34]. As the follicle grows and the antral cavity becomes filled with follicular fluid, VEGF production increases and the follicular fluid becomes rich in VEGF [117, 118]. These aspects have been confirmed by increasing of the VEGF ligand (mRNA and protein) with the proliferation of microvessels, progression of gestation, and ovarian development [34].

The role of VEGF in promoting the primary to secondary follicle transition has been demonstrated in vitro during culture of fetal bovine ovarian tissue [34]. In goats, VEGF has been shown to be crucial to the in vitro growth of preantral follicles and their oocytes enclosed in ovarian tissue [119], and to meiosis progression during the maturation of oocytes grown from secondary follicles cultured in vitro [42]. Recently, we have demonstrated that VEGF increases antrum formation and follicular growth rate after in vitro culture of bovine preantral follicles (Araújo et al., unpublished observations). Taken together, these results lead us to believe that VEGF may be an excellent constituent for the in vitro culture media for bovine secondary follicles.

### Hormones

There seems to exist an overall consensus that preantral follicles can develop in the absence of gonadotropins. However, the use of gonadotropins for in vitro culture has been important for obtaining optimal development of preantral follicles. Treatment of large, isolated preantral follicles with FSH stimulated granulosa cell proliferation and antrum formation [37–39, 65, 79]. Moreover, hormones such as FSH [38, 39, 79], and activin stimulated steroidogenesis in bovine isolated preantral follicles [22, 39, 79]. The following sections will describe only the hormones which have been used in in vitro culture systems for bovine preantral follicles and ovarian cells.

#### Activin

Activin is a proteic hormone that enhances FSH biosynthesis and secretion. Activin and its receptor are expressed on theca and granulosa cells, and oocytes of bovine preantral follicles [120]. It is composed of two beta subunits, A and B, and exists as a homo- (A and B) or heterodimer (AB) with activin-A as the predominant isoform. Activins are involved in primordial follicle activation in vitro [121], preantral follicle development [120, 122–124], granulosa cell proliferation, antral cavity formation, maintenance of normal oocyte morphology, and interactions between the oocyte and granulosa cells [22]. Polarized expression of cell contact interactions promoted by activin supports ongoing folliculogenesis, which is characterized by increased peripheral granulosa cell adhesion to the basement membrane and retention of adhesion at the surface of the ZP [22].

Although activin has caused a significant increase in the size of follicles and estradiol concentrations in immature mice, in adult mice it did not change the follicle diameter and completely blocked the action of FSH on both follicle diameter and estradiol concentration [125]. In sheep, the activin promoted preantral follicle and oocyte growth in vitro, but did not accelerate follicle differentiation or have any effect on antrum formation or follicle survival. Moreover, activin and FSH interacted positively to stimulate the follicle growth and granulosa proliferation of bovine preantral follicles [120]. All these results indicate that activin has a paracrine role through proliferative and cytodifferentiative action on granulosa cells and that its action is age and FSH dependent.

#### Follicle-stimulating hormone (FSH)

Gonadotropins seem to be important for the optimal development of preantral follicles in vitro. FSH in particular is considered a critical hormone for the survival of large secondary follicles [126] and its receptor (FSH-R) has been observed in granulosa cells of bovine preantral follicles (primary and secondary; [110]). In vitro culture of bovine isolated preantral follicles with FSH stimulated an increase in follicular diameter [65, 120], granulosa cell proliferation by BrdU-label [120], and progesterone [65] and estradiol secretion [22]. Moreover, FSH maintained normal oocyte morphology and interactions between the oocyte and granulosa cells after in vitro culture of bovine secondary follicles [22]. Wandji et al. [65] observed that large preantral follicles (150 to 220 μm) produced more progesterone in response to FSH than smaller (60 to 179 μm) preantral follicles. These findings indicated that the responsiveness to FSH increases as the bovine follicles develop.

#### Growth hormone (GH)

Among metabolism-related endocrine factors, GH has been shown to be a crucial factor for follicular development in the mammalian ovary [127]. Both fetal and adult bovine ovaries revealed distinct amounts of GH receptor (GHR) and its transcript in the oocytes of primordial and primary follicles, as well as mRNA for GHR in secondary and tertiary follicles. These results support the concept that GHR is involved in the development and differentiation of primordial follicles in both prenatal and postnatal life [128].

In vivo, GH may stimulate specific follicle populations selectively. GH inhibited the development of preovulatory follicles and stimulated the growth of the second-largest follicle in heifers [129]. GHR immunoreactivity and mRNA encoding GHR in granulosa cells, theca cells and luteal cells of the bovine ovary [128] suggests the GH action by means of the detection of GH binding activity. Moreover, GHR expression increases in the granulosa cells when the follicles become estrogen-active, even when compared to preovulatory follicles [128]. Thus, the increase in GHR expression in these follicles may be regulated by estradiol [128]. The negative interaction between GH and estradiol during later folliculogenesis seems to be true; however, this effect seems to be positive during early folliculogenesis, since the addition of GH to the culture medium of bovine preantral follicle increased the estradiol concentrations (Araújo et al., unpublished observations). In addition, bovine granulosa cells' expression of mRNA for GHR was stimulated in vitro by FSH [127]. Also, GH has been shown to enhance cell proliferation and steroidogenesis of cultured granulosa cells in cattle [130], suggesting an important role for GH in the regulation of granulosa cell proliferation and follicular growth.

#### Insulin

Among the endocrine factors, insulin is a crucial hormone for follicular development [127], granulosa cell function, and ovulation [131]. Additionally, insulin may regulate various intracellular processes in the follicle, such as amino acid transport, lipid metabolism, gene transcription, and protein synthesis [132]. Insulin acts through its own receptor, which first appears in the granulosa cells of small bovine antral follicles [131]. Insulin receptor is widely distributed throughout all ovarian compartments, including granulosa and theca cells and stromal tissue [127, 131]. In addition, the concentrations of insulin in follicular fluid are constant at all follicular developmental stages [127].

Infusion of insulin in beef heifers increased the diameter of the dominant follicle [133]. High levels of insulin receptor mRNA expression in granulosa cells of preovulatory follicles seem to be necessary for development of the ovulatory stage [127]. The presence of insulin receptor in small antral follicles, together with its absence in preantral follicles, indicates the involvement of insulin, and the acquisition of its receptor, during early follicular growth in bovine [131]. This hypothesis has been supported by the fact that increased dietary intake of insulin was associated with recruitment of small follicles (<4 mm), but did not affect follicle selection (medium: 4-8 mm) or dominant (large: >8 mm) follicles [134].

In vitro, insulin has been shown to be essential for follicle culture. Absence of insulin in the culture medium resulted in follicle degeneration [94]. Gutierrez et al. [20] demonstrated that bovine preantral follicles grew for a long period in culture, even in the absence of tropic hormones, but in the presence of insulin. Considering that type 1 IGF-1 receptor is present in oocytes and granulosa and theca cells of bovine preantral follicles [111], and that insulin competes with the IGF receptors, the follicular growth effect was probably promoted by the interaction of insulin and the type 1 IGF-1 receptor.

#### Estrogens and androgens

Steroid hormones have been shown to be involved in growth of bovine preantral follicles in vitro (estradiol: [135]; testosterone: [80]. Moreover, its receptors have been reported to be present in bovine ovaries [80]. Addition of estradiol in vitro increased follicular diameter of bovine isolated preantral follicles without affecting the proliferation of granulosa cells [135]. Furthermore, a combined action of insulin, estradiol, and human chorionic gonadotropin (hCG) promoted granulosa cell proliferation, as well as the growth of primordial follicles into primary follicles after 48 h of in vitro culture of bovine ovarian cortical tissue [68]. In addition, treatment with estradiol in vivo stimulated activation of primordial follicles, and its combination with bovine somatotropin (bST) decreased the rate of atresia of primary follicles [136]. Moreover, testosterone was reported to promote growth of primary to secondary follicles after in vitro culture of bovine cortical fragments [80]. Using immunohistochemistry to detect androgen receptors (AR) the previous authors also demonstrated that granulosa and theca cells of only secondary and tertiary follicles exhibited AR staining. Furthermore, estradiol production was substantially reduced when an anti-androgen antibody was used during in vitro culture of mouse preantral follicles [137]; however, this effect was neutralized with androstenedione, which significantly increased estradiol production.

### Final considerations

Several studies of bovine folliculogenesis have examined the aspects of in vitro follicular development. However, it is still not clear which culture medium needs to be used to culture bovine preantral follicles, as well as which growth factors and hormones could influence follicular development. Moreover, it will be important to have an optimum and standard culture system for bovine follicles, either using two- or three-dimensional approaches. A culture system to be selected needs to affect positively follicular morphology, survival, proliferation, steroidogenesis, and gene expression. Furthermore, it is important to reevaluate the effect of growth factors and/or hormones on follicular growth. The follicle microenvironment must be considered, as well as the role of growth factors and hormones and their respective signaling pathways during in vitro follicular development. Additionally, factors such as age (immature or adult) of the animals, follicular category (early or late preantral follicles, or antral follicles) to be used, and system of in vitro follicular culture (two- or three-dimensional) should be considered very important sources of data variation.

## Abbreviations

BMPs: Bone morphogenetic proteins
BrdU-label: Bromodeoxyuridine
DNAse: Deoxyribonuclease
FGFs: Fibroblast growth factors
flk-1 or VEGFR-2: Kinase domain receptor (VEGF receptor 2)
flt-1 or VEGFR-1: Fms-like tyrosine kinase-1 (VEGF receptor 1)
FSH: Follicle-stimulating hormone
GDFs: Growth and differentiation factors
GH: Growth hormone
GHR: GH receptor
IGFBP-2: IGF binding protein-2
IGFs: Insulin-like growth factors
LH: Luteinizing hormone
α-MEM: Minimum essential medium alpha modification
MMP-9: Matrix metalloproteinases-9
OPU: Ovum pick-up technique
TCM-199: Tissue culture medium-199
TGF-β: Transforming growth factor β
VEGF: Vascular endothelial growth factor
ZP: Zona pellucida
2D: Two-dimensional system
3D: Three-dimensional system.
